# Biomarkers after Controlled Inhalation Exposure to Exhaust from Hydrogenated Vegetable Oil (HVO)

**DOI:** 10.3390/ijerph18126492

**Published:** 2021-06-16

**Authors:** Annette M. Krais, Julie Y. Essig, Louise Gren, Carolina Vogs, Eva Assarsson, Katrin Dierschke, Jörn Nielsen, Bo Strandberg, Joakim Pagels, Karin Broberg, Christian H. Lindh, Anders Gudmundsson, Aneta Wierzbicka

**Affiliations:** 1 Division of Occupational and Environmental Medicine, Department of Laboratory Medicine, Lund University, SE-22363 Lund, Sweden; yona.essig@y-es.de (J.Y.E.); eva.assarsson@med.lu.se (E.A.); katrin.dierschke@skane.se (K.D.); jorn-nielsen@telia.com (J.N.); Bo.Strandberg@skane.se (B.S.); karin.broberg@med.lu.se (K.B.); christian.lindh@med.lu.se (C.H.L.); 2 Division of Ergonomics and Aerosol Technology, Department of Design Sciences, Lund University, SE-22100 Lund, Sweden; louise.gren@design.lth.se (L.G.); joakim.pagels@design.lth.se (J.P.); anders.gudmundsson@design.lth.se (A.G.); aneta.wierzbicka@design.lth.se (A.W.); 3 NanoLund, Center for Nanoscience, Lund University, SE-22100 Lund, Sweden; 4 Department of Biomedical Sciences and Veterinary Public Health, Swedish University of Agricultural Sciences, SE-75007 Uppsala, Sweden; carolina.vogs@slu.se

**Keywords:** renewable diesel, HVO, exposure studies, biomarkers, aerosol, lipid peroxidation

## Abstract

Hydrogenated vegetable oil (HVO) is a renewable diesel fuel used to replace petroleum diesel. The organic compounds in HVO are poorly characterized; therefore, toxicological properties could be different from petroleum diesel exhaust. The aim of this study was to evaluate the exposure and effective biomarkers in 18 individuals after short-term (3 h) exposure to HVO exhaust and petroleum diesel exhaust fumes. Liquid chromatography tandem mass spectrometry was used to analyze urinary biomarkers. A proximity extension assay was used for the measurement of inflammatory proteins in plasma samples. Short-term (3 h) exposure to HVO exhaust (PM_1_ ~1 µg/m^3^ and ~90 µg/m^3^ for vehicles with and without exhaust aftertreatment systems, respectively) did not increase any exposure biomarker, whereas petroleum diesel exhaust (PM_1_ ~300 µg/m^3^) increased urinary 4-MHA, a biomarker for *p*-xylene. HVO exhaust from the vehicle without exhaust aftertreatment system increased urinary 4-HNE-MA, a biomarker for lipid peroxidation, from 64 ng/mL urine (before exposure) to 141 ng/mL (24 h after exposure, *p* < 0.001). There was no differential expression of plasma inflammatory proteins between the HVO exhaust and control exposure group. In conclusion, short-term exposure to low concentrations of HVO exhaust did not increase urinary exposure biomarkers, but caused a slight increase in lipid peroxidation associated with the particle fraction.

## 1. Introduction

Exposure to petroleum diesel engine exhaust is associated with an increased risk of lung cancer, as classified by the International Agency for Research on Cancer (IARC) [1]. The European Union has set a new occupational exposure limit (OEL) of 50 µg/m^3^ for diesel engine exhaust emissions, measured as elemental carbon (EC) [2]. This limit value is a complement to existing OELs of nitrogen oxides (NOx) and polycyclic aromatic hydrocarbons (PAHs), and it will become effective in general occupational health environments from 2023.

Renewable diesel fuels have been developed to replace the use of petroleum diesel, thus aiming to reduce climate impacts. Examples are hydrotreated vegetable oil (HVO) and biodiesels of fatty acid methyl ester (FAME), which have the potential to reduce the net emissions of carbon dioxide (CO_2_) and particulate matter (PM) [3]. Compared to petroleum diesel, HVO exhaust fumes are generally found to be reduced in PM [4,5,6] and PAHs [3], but increased in straight chain alkanes [3]. The use of renewable diesel is expected to increase over the coming years, with HVO being the fuel predominantly used to replace petroleum diesel in full substitution, due to its greater engine compatibility compared to FAME. However, the exact composition of organic compounds present in HVO exhaust is still unclear, because new compounds could be formed during the combustion process. Therefore, toxicological properties could be different from petroleum diesel exhaust.

Although a few controlled human exposure studies of FAME fuels exist [7,8], no such studies have been performed with HVO fuels, to the best of the authors’ knowledge. A recent review reported several in vitro studies linking HVO exhaust to oxidative stress, inflammation and genotoxicity, whereas animal studies did not indicate genotoxicity in lung tissue [9]. Bendtsen et al. found that the toxicity of HVO and petroleum diesel in mice were similar, but depended largely on engine conditions; higher reactive oxygen species (ROS) formation and more DNA strand breaks were linked to engine conditions that favored high combustion temperatures [5,10].

The aim of this study was to investigate the human response to short-term (3 h) inhalation exposure to HVO exhaust, and to possibly detect specific exposure and effect biomarkers in human urine. Exposures included particle-free air, salt aerosol as particle control, and HVO exhaust from two different modern non-road vehicles, with and without aftertreatment systems [11]. In 18 participants of the study, urinary biomarkers for HVO exhaust were explored, such as metabolites of PAHs and other volatile organic compounds (VOCs), as well as 8-oxo-2’-deoxyguanosine (8-oxodG), a marker for DNA damage; 4-hydroxynonenal mercapturic acid (4-HNE-MA), a marker for lipid peroxidation; and plasma expression of 92 inflammatory-related proteins. For comparison, these biomarkers were also analyzed in samples from a similar short-term (3 h) human exposure study with petroleum diesel exhaust [12].

## 2. Materials and Methods

This study is based on an analysis of biomarkers of urine samples from two exposure studies (HVO and petroleum diesel). Calculations for the total excretion of metabolites and the expression of inflammatory proteins using the proximity extension assay were performed in samples from the HVO exposure study only, due to a lack of samples from the petroleum diesel study.

### 2.1. Chemicals and Reagents

Toluene and acetonitrile (LC-MS grade) were purchased from Merck (Darmstadt, Germany), and formic acid was from Sigma-Aldrich (St Louis, MO, USA). β-Glucuronidase (*Escherichia coli*) was obtained from Roche Diagnostics (Mannheim, Germany). Water was produced by the Milli-Q Integral 5 system, Millipore (Billerica, MA, USA). The analytical standard 8-oxodG was purchased from Sigma-Aldrich, and ^15^N_5_-8-oxodG was from Cambridge Isotope Laboratories Inc. (Tewksbury, MA, USA). All other chemical standards and deuterium-labeled internal standards were purchased from Toronto Research Chemicals (Toronto, ON, Canada). NaCl (>99.5%) was purchased from Sigma-Aldrich and ultrapure water was from VWR Chemicals (Darmstadt, Germany).

### 2.2. Exposure Study with HVO Exhaust

The study was approved by the Swedish Ethical Review Authority (registration Dnr: 2019-03320) and performed in accordance with the Declaration of Helsinki, including obtaining written informed consent from all subjects. The study was performed in 2019. Before exposure, all the participants underwent a medical examination, and all participants’ lung function values were classified as normal [11]. Nineteen volunteers were selected as fulfilling the inclusion criteria for the study: men or non-pregnant women, 20−65 years old, no known lung disease, no asthma diagnosis, normal standard ECG reading, no medication that would affect monitored parameters, and a non-smoker for the last three years [11]. In total, 19 volunteers (9 females, 10 males) participated, but only 18 of them (9 females, 9 males) completed all exposures and biological samplings.

In total, four exposure scenarios were included: (i) blank exposure with filtered air (FA); (ii) particle control with dry NaCl salt particles (Salt_PM_); (iii) HVO exhaust containing mainly NOx (HVO_NOx_); and (iv) HVO exhaust containing PM, NOx, and organics (HVO_PM+NOx_) [11]. Each of the four exposure scenarios was carried out with a maximum of four persons sitting in the chamber for 3 h, with a washout period of at least seven days between the different exposure scenarios. The exposures took place in a 22 m^3^ stainless steel chamber with controlled humidity, temperature, and ventilation. All details are described by Gren et al. [11]. The average exposure concentrations are summarized in Table S1.

### 2.3. Exposure Study with Petroleum Diesel Exhaust

The study was approved by the Regional Ethical Committee in Lund (Dnr 2009/460), and performed in accordance with the Declaration of Helsinki, including obtaining written informed consent from all subjects. The study was performed in 2009–2010. Eighteen healthy, non-smoking volunteers (9 males and 9 females), aged 40−66 years, were exposed to petroleum diesel emissions with a high concentration of diesel exhaust particles (276 ± 27 µg/m^3^ PM_1_) and filtered air (FA) for control exposures. The diesel exhaust was generated by an idling (900 rpm) Volkswagen Passat TDI (-98, 1900 cm^3^, 81 kW). Test subjects (three at each exposure, relaxed sitting) were exposed to each exposure scenario for 3 h, with at least a one-week interval between each scenario. The PM_1_ (<1 µm) mass concentrations were determined with a tapered element oscillating microbalance (TEOM, model 1400a, Thermo Fisher Scientific Inc., Franklin, MA, USA). A PM_1_ cyclone and a Nafion dryer were installed upstream of the TEOM. Details of the method are described by Wierzbicka et al. [12]. The average exposure concentrations are summarized in Table S1.

### 2.4. Aerosol Generation

The HVO exhaust was generated with two types of modern non-road vehicles manufactured in 2019 and complying with current EU emission standards of non-road mobile machinery. One vehicle (HVO_PM+NOx_) complied with emission standard Stage IIIa (23 kW) and did not have any external exhaust aftertreatment. The other vehicle complied with Stage V (55.4 kW), and had a diesel oxidation catalyst (DOC) and a diesel particulate filter (DPF) (HVO_NOx_). Both vehicles operated with 100% HVO fuel [11]. The use of the two vehicles allowed for a comparison of exposure to an exhaust consisting of NOx, particles and organics, with an exhaust dominated by NOx with minimal amounts of other pollutants.

Chemical-grade NaCl was dissolved in ultrapure water and nebulized using a constant output atomizer model 3076 (TSI Inc. Shoreview, MN, USA) and custom-built nebulizer. The aerosol was dried with a dilution flow of HEPA-filtered air in a 10 L steel chamber. The relative humidity (RH) was kept below 30% in this step (except during one exposure that had <40% RH), to ensure that the salt aerosol was in dry/crystalline form.

Filtered air was used for the blank reference exposure. The air was passed through a HEPA filter and an active carbon filter. The particle number concentration was on average 71 ± 43 #/cm^3^, and the average VOC concentration was <10 ppb. The average exposure concentrations and particle characteristics are summarized in Table S1. Full details of the exposures and exhaust measurements have been described earlier, for HVO exhaust [11] and petroleum diesel exhaust [12].

### 2.5. Analysis of Airborne PAHs and BTEX

Air samples for PAH analysis were collected during the entire duration of the exposure (180 min) on Teflo air sampling membranes (PTFE, 37 mm diameter, 2 µm pore size, Pall Corp., Port Washington, NY, USA) followed by XAD-2 tubes (SKC Inc., Dorset, UK). Particle filters and XAD tubes were extracted using dichloromethane analyzed for 32 native PAHs (including the 16 U.S. EPA priority PAHs) and 16 alkylated species. Target compounds were separated on an Agilent 5975C mass spectrometer (MS) coupled to a 7890A gas chromatograph (GC, Agilent Technologies, Santa Clara, CA, USA) [11]. Analyses of benzene, toluene, ethyl benzene, *m* + *p* xylene, and *o*-xylene (BTEX) were performed by the Swedish Environmental Institute (IVL). The samples were collected on Tenax^®^ TA thermal desorption tubes (SKC Inc., Dorset, UK) and analyzed by thermal desorption GC-MS. Target compounds were separated on a non-polar analytical mass spectrometer (TraceGold, TG-1MS, Thermo Fisher Scientific Inc., Franklin, MA, USA) coupled to an ISQ LT (Thermo Fisher Scientific Inc., Franklin, MA, USA) [11].

### 2.6. Calculation of Inhaled Dose

The total inhaled dose of PAHs and BTEX during the 3 h exposure was estimated individually for each participant with Equation (1):

(1) $$\mathrm{Inhaled}\;\mathrm{dose}=V_{t}\;\times \;B_{f}\;\times \;t\;\times \;[P]$$

where V_t_ is the individual tidal volume (L) measured by forced oscillation technique (FOT, Tremoflo, THORASYS, Thoracic Medical System Inc., Montreal, QC Canada), B_f_ is the individual breathing frequency (min^−1^) measured by a Noxturnal 5.1 breathing belt (NOX Medical, ResMed, Kista, Sweden), t is the total exposure time (180 min), and [P] is the average exposure concentration of the respective gas phase compound (PAH, BTEX). The inhaled and lung-deposited particle doses of HVO_PM+NOx_ are discussed in Gren et al. [11].

### 2.7. Urinary Metabolites LC-MS/MS

Sample Collection

For all exposure scenarios of the HVO exposure study, urine samples were collected immediately before each exposure (t = 0 h) and immediately after exposure (t = 3 h). Subsequently, urine was collected for a maximum of 24 h. Participants were asked to collect all urine for 24 h at regular intervals, i.e., every hour. Pilot studies performed in our laboratory showed that excreted concentrations of metabolites were below the detection limit even at 24 h after exposure. For the exposure study with petroleum diesel, only spot urine samples were collected: before each exposure (t = 0 h), immediately after exposure (t = 3 h), and the next morning (t = 24 h). Urine volumes and urine density were measured before aliquoting the urine samples. Samples were stored at −20 °C prior to analysis.

#### 2.7.1. Analysis of PAH Metabolites

Sample Preparation

Samples comprising 0.2 mL of urine were transferred to glass inserts (Teknolab Sorbent, Kungsbacka, Sweden) of a 96-well Rittner plate (Teknolab Sorbent), and 0.1 mL of ammonium acetate (pH 6.5) and 0.01 mL of β-glucuronidase (*Escherichia coli*) were added. The solution was incubated for 30 min at 37 °C. Afterwards, 0.025 mL of a 50:50 (*v*/*v*) water:acetonitrile solution and 0.025 mL of deuterium-labeled internal standards (D_7_-2-Nap, D_8_-2-OH-Flu, D_9_-1-OH-Phe, D_9_-2-OH-Phe, D_9_-4-OH-Phe, D_9_-1-OH-Pyr) were added (at final concentrations of 5 ng/mL). The plates were centrifuged for 10 min at 3000 rpm, prior to injection (3 μL).

LC-MS/MS Instrumentation and Conditions

Samples were analyzed for 2-OH-Nap, 1-OH-Phe, 2,3-OH-Phe, 4-OH-Phe, 2,3-OH-Flu and 1-OH-Pyr. A C18 column (2.1 mm i.d. × 50 mm; Grace Genesis Lighting, Sunrise, FL, USA) was used before the injector to reduce the interference of contaminants during the mobile phase. A Luna C18(2) HST column (2.5 µm, 100 Å, 2.1 mm i.d. × 100 mm, Phenomenex, Torrance, CA, USA) was used for separation, and the mobile phases were Milli-Q water (A) and acetonitrile:methanol (50:50, B). The samples were analyzed on a Shimadzu UFLC system (Shimadzu Corp., Kyoto, Japan) coupled to a QTRAP 5500 (triple quadrupole linear ion trap mass spectrometer) equipped with a TurboIon Spray source (AB Sciex, Framingham, MA, USA). The gradient was linearly increased from 5% B to 80% B within 6 min, then to 95% B within 10 s and held there for 1 min 40 s, before decreasing back to 5% B within 10 s and holding at 5% B for 2 min. 2-OH-Phe and 3-OH-Phe, as well as 2-OH-Flu and 3-OH-Flu could not be separated, and were therefore analyzed as single peaks (∑2,3-OH-Phe and ∑2,3-OH-Flu, respectively). Table S2 presents the ion transitions for all quantified compounds and internal standards (ISs), as well as collision energies (CEs) and retention times (RTs). The limit of detection (LOD) was calculated from blank chemical samples and defined as three times the standard deviation of the concentration corresponding to the peak at the same retention time as the individual compounds. All PAH metabolite concentrations were adjusted to urinary density (ng/mL urine). Urine density was measured using a digital refractometer (Refractometer 30PX, Mettler Toledo, Columbus, OH, USA). Details of the method have been published previously [13].

#### 2.7.2. Analysis of Other Urinary Biomarkers

Sample Preparation

Samples comprising 0.2 mL of urine were transferred to glass inserts (Teknolab Sorbent) of a 96-well Rittner plate (Teknolab Sorbent), and 0.1 mL of ammonium acetate (pH 6.5) and 0.01 mL of β-glucuronidase were added. The solution was incubated for 30 min at 37 °C. Afterwards, 0.025 mL of a 50:50 (*v*/*v*) water:acetonitrile solution and 0.025 mL of isotope-labelled internal standards (D_7_-4-MHA, D_4_-BMA, D_5_-PMA, ^13^C_4_-4-MU, D_6_-3-HPMA, ^15^N_5_-8-oxodG) were added (final concentrations of 5 ng/mL). The plates were centrifuged for 10 min at 3000 rpm, prior to injection.

LC-MS/MS Instrumentation and Conditions

Samples were analyzed for 4-MHA, BMA, PMA, 4-MU, 3-HPMA, 4-HNE-MA and 8-oxodG. A C18 column (2.1 mm i.d. × 50 mm; Grace Genesis Lighting) was used before the injector, and a Restek UltaAQ C18 column (3 µm, 4.6 mm i.d. × 100 mm, Restek, Philadelphia, PA, USA) was used for separation. The samples were analyzed on a Shimadzu UFLC system (Shimadzu Corp.) coupled to a QTRAP 5500 (triple quadrupole linear ion trap mass spectrometer) equipped with a TurboIon Spray source (AB Sciex). Solvents were Milli-Q water (A) and acetonitrile (B), containing 0.1% formic acid. Subsequently, 4-MU, BMA, PMA and 8-oxodG were analyzed with a flow of 0.6 mL/min and an injection volume of 3 µL. The gradient was linearly increased from 5% B to 95% B within 4 min and held there for 1 min, before decreasing back to 5% B within 10 s and holding at 5% B for 1 min. Then, 4-MHA, 3-HPMA and 4-HNE-MA were analyzed using a flow of 1.2 mL/min and an injection volume of only 0.2 µL. Here, the gradient was linearly increased from 5% B to 95% B within 3 min and held there for 2 min, before decreasing back to 5% B within 10 s and holding at 5% B for 1 min. All concentrations were adjusted to urinary density (ng/mL urine). Table S2 presents the ion transitions and other parameters for detection of the quantified compounds.

### 2.8. Pharmacokinetic Evaluation

For the HVO exposure study, participants were asked to collect all urine up to 24 h after exposure, resulting in 6−10 urine samples per participant, with volumes ranging from 100 mL to 500 mL. The total excreted concentrations of urinary metabolites in these samples were calculated and expressed as area under the excretion curve (AUC). The AUC was determined using the trapezoidal method [14]. Here, the AUC (µg × min/mL) was calculated for each subject and each exposure scenario to determine the individual total metabolite excretion over a time course of 24 h according to Equation (2):

(2) $$\mathrm{AUC}=\overset{t=0}{\underset{t=24}{\sum }}\frac{\frac{1}{2}\;\times \;(C_{i}+C_{i+1})}{\left(t_{i+1}-t_{i}\right)}$$

where C_i_ and C_i+1_ are the metabolite concentration at time points t_i_ and t_i+1_, respectively, with t_i_ < t_i+1_.

The average and median AUC and its standard deviation were calculated to compare the metabolite exposures between the four different exposure scenarios. The coefficient of variance (CV) was calculated as the ratio of the standard deviation to the average AUC. For the exposure study on petroleum diesel, no such calculations were possible, because only spot urine samples were collected, while in the HVO exposure study, the total excreted volume of urine was collected for 24 h.

### 2.9. Expression of Inflammatory Proteins

Plasma samples were collected using 4 mL Becton Dickinson vacutainer K2 EDTA tubes (BD, Belliver Industrial Estate, Plymouth, UK). Proteins (N = 92) related to inflammatory processes were measured using proximity extension assay (PEA) technology (Olink Proteomics AB, Uppsala, Sweden) according to the manufacturer’s instructions. The PEA protocol [15] enables 92 analytes to be analyzed simultaneously, using 1 µL of each sample. Briefly, pairs of oligonucleotide-labeled antibody probes bind to their targeted protein, and if the two probes are brought in close proximity, the oligonucleotides will hybridize in a pair-wise manner. The addition of a DNA polymerase leads to a proximity-dependent DNA polymerization event, generating a unique PCR target sequence. The resulting DNA sequence was subsequently detected and quantified using a microfluidic real-time PCR instrument (Biomark HD, Fluidigm Corp., San Francisco, CA, USA). Data were then quality-controlled and normalized using an internal extension control and an inter-plate control, to adjust for intra- and inter-run variation. The final assay read-outs are presented in normalized protein expression (NPX) values, which are an arbitrary unit on a log2 scale where a high value corresponds to a higher protein expression. All assay validation data (detection limits, intra- and inter-assay precision data, etc.) are available on the manufacturer’s website (www.olink.com).

### 2.10. Statistical Analysis

Statistical comparisons were performed with Prism Graph-Pad Software (version 8.2.0, San Diego, CA, USA) using the Wilcoxon signed rank test or Mann–Whitney U test when appropriate (* *p* < 0.05, ** *p* < 0.01, *** *p* < 0.001).

## 3. Results and Discussion

### 3.1. Urinary PAH Metabolites

**Figure 1:**
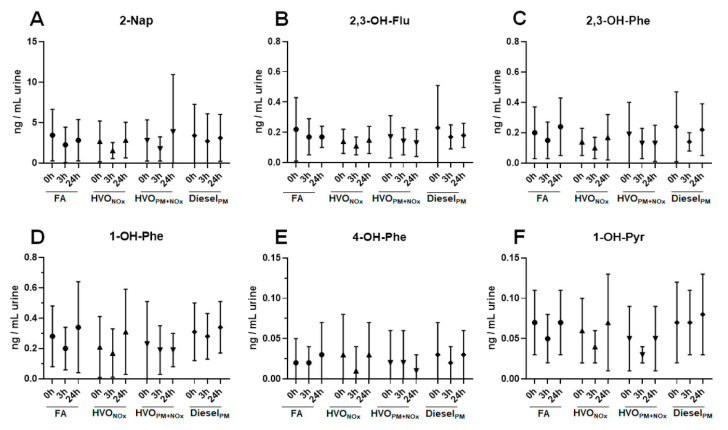
Urinary metabolites of PAHs after different exposure scenarios. Average and standard deviation of the urinary metabolites 2-Nap (**A**), 2,3-OH-Flu (**B**), 2,3-OH-Phe (**C**), 1-OH-Phe (**D**), 4-OH-Phe (**E**), and 1-OH-Pyr (**F**) from 18 volunteers after exposure to: filtered air (FA), HVO exhaust from a modern non-road vehicle with exhaust aftertreatment (HVO_NOx_) and without exhaust aftertreatment (HVO_PM+NOx_), as well as petroleum diesel exhaust (Diesel_PM_). Statistical analysis was performed using Wilcoxon signed rank test. No significant difference (*p* < 0.05) was observed between any of the exposure scenarios, including FA and the particle control (Salt_PM_). All data are presented in Table S3.

Figure 1 and Table S3 show the concentrations of PAH metabolites in spot urine samples (t = 0h, 3h, and 24h) after different exposure scenarios. The most abundant PAH metabolite was 2-napthol (2-Nap), with average concentrations ranging from 1.6 to 3.9 ng/mL urine between exposure scenarios. Other PAH metabolites, including 2,3-hydroxyfluorene (2,3-OH-Flu), 2,3-hydroxyphenanthrene (2,3-OH-Phe), 1-hydroxyphenanthrene (1-OH-Phe), 4-hydroxyphenanthrene (4-OH-Phe), and 1-hydroxypyrene (1-OH-Pyr), were present in concentrations below 0.5 ng/mL urine. For comparison, urinary PAH metabolites collected after a similar human exposure study with petroleum diesel exhaust (Diesel_PM_), performed in 2009–2010 [12] were analyzed. Diesel_PM_ exhaust contained 3-fold higher concentrations of PM_1_ compared to HVO_PM+NOx_ (276 µg/m^3^ and 93 µg/m^3^, respectively), and 9-fold higher concentrations of gas phase PAHs (7.5 × 10^3^ ng/m^3^ and 850 ng/m^3^, respectively), while particle-bound PAHs were similar (60 ng/m^3^ and 43 ng/m^3^, respectively) (Table S1).

No difference in PAH metabolite concentrations was observed between different time points (0 h, 3 h, or 24 h after exposure), or different exposure scenarios (FA, Salt_PM_, HVO_NOx_, HVO_PM+NOx,_ and Diesel_PM_) (Figure 1 and Table S3). Two types of control exposures were included in this study: filtered air (FA) and a salt aerosol that was used as particle control (Salt_PM_). No significant difference of the urinary metabolites was detected between FA and Salt_PM_; therefore, data for Salt_PM_ are only shown in Table S3. Our results confirm an earlier report that did not find significantly altered levels of 14 urinary (unmetabolized) PAHs after the same controlled exposure study with Diesel _PM_ exhaust [16].

In the body, PAHs are metabolized and excreted rapidly in the urine. Depending on the individual PAH, the maximal concentrations are reached within different times, from 3.1 h (1-Nap) to 5.5 h (1-OH-Pyr) [17]. It was thus reasonable to expect higher levels of PAH metabolites right after exposure (t = 3 h), at least for the volatile 2-Nap, but no alteration in PAH levels was observed, even after exposure to the PAH-rich Diesel_PM_ exhaust. For the four exposure scenarios of the HVO exposure study, all urine volumes were sampled continuously up to 24 h after exposure. No alterations in PAH metabolite levels were observed in any of the urine samples of the 18 volunteers (data not shown).

Concentrations of PAH metabolites were in the same range as those typically found in the general population. A study on young adults in Sweden reported similar urinary concentrations (median values): 0.22 ng/mL (2,3-OH-Flu), 0.18 ng/mL (2,3-OH-Phe), 0.16 ng/mL (1-OH-Phe), 0.013 ng/mL (4-OH-Phe), and 0.066 ng/mL (1-OH-Pyr) [18]. Grainger et al. measured slightly higher average concentrations in the general US population: 4.7− 8.4 ng/mL (2-OH-Flu), 2.5−4.5 ng/mL (3-OH-Flu), 0.7−0.9 ng/mL (2-OH-Phe), 0.6−0.9 ng/mL (3-OH-Phe), and 0.9−1.4 ng/mL (1-OH-Pyr) [19].

Our results suggest that PAH air concentrations in our exposure studies did not exceed normal background exposure in the general population. Besides combustion processes, PAHs can result from gas usage, cooking (frying and oil combustion), and smoking, as well as candle and incense burning [20]. Air concentrations of gas phase PAHs in the HVO exposure study were low, with maximal concentrations of 1 µg/m^3^ for PAHs during the HVO_PM+NOx_ exposure (Table S1), and higher in the petroleum diesel study, with 7.5 µg/m^3^ of PAHs. Several studies from Stockholm reported lower outdoor levels, with 1.38 ng/m^3^ [21], and 6.90 ng/m^3^ (gas phase) and 1.27 ng/m^3^ (particle phase) [22]. A recent study from the US EPA reported outdoor air concentrations of 45.4 ± 57.1 ng/m^3^ [23]. Due to differences in total number of PAHs, a direct comparison of sum values is difficult. Additionally, no specific guideline value can be recommended for air concentrations, as PAHs are typically present in complex mixtures [24]. Risk concentrations for PAHs have been estimated with 3 µg/m^3^ (low molecular weight PAHs) [25] and 6 µg/m^3^ (medium molecular weight PAHs) [24]. This value of 6 µg/m^3^ was reached after the short-term (3 h) exposure to petroleum diesel, but not for any of the HVO exposure scenarios.

### 3.2. Urinary VOC Metabolites

VOCs commonly linked to traffic-related air pollution were assessed with the following biomarkers: 4-methylhippuric acid (4-MHA), a marker of *p*-xylene exposure; *N*-acetyl-*S*-(phenyl)-L-cysteine (PMA), a marker of benzene exposure; *N*-acetyl-*S*-(benzyl)-L-cysteine (BMA), a marker of toluene exposure; 3-hydroxypropyl mercapturic acid (3-HPMA), a marker for acrolein; and 4-methylumbilliferone (4-MU), a putative marker for diesel exhaust.

**Figure 2:**
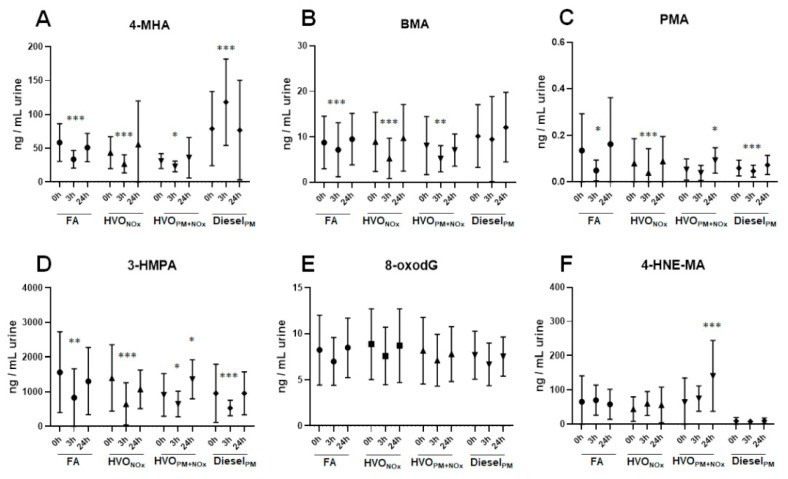
Urinary VOC metabolites, as well as 8oxodG and 4-HNE-MA, after different exposure scenarios. Average and standard deviation of the urinary biomarkers 4-MHA (**A**), BMA (**B**), PMA (**C**), 3-HPMA (**D**), 8-oxodG (**E**), and 4-HNE-MA (**F**) from 18 volunteers after exposure to: filtered air (FA), HVO exhaust from a modern non-road vehicle with exhaust aftertreatment (HVO_NOx_) and without exhaust aftertreatment (HVO_PM+NOx_), as well as petroleum diesel exhaust (Diesel_PM_). Statistical analysis was performed using Wilcoxon signed rank test (* *p* < 0.05, ** *p* < 0.01, *** *p* < 0.001, different from t = 0 h). No significant difference was observed between FA and the particle control exposure (Salt_PM_). All data are presented in Table S3.

Figure 2A−D and Table S3 show the concentrations of VOC biomarkers in spot urine samples (t = 0 h, 3 h, and 24 h) after different exposure scenarios. 3-HPMA was found in high concentrations in all samples, with average concentrations ranging from 533 to 1564 ng/mL urine, followed by 4-MHA (23−118 ng/mL urine) and BMA (5.2−11 ng/mL urine). PMA and 4-MU were detected in very low concentrations (below 0.4 ng/mL and 1.6 ng/mL, respectively). Most urinary VOC biomarkers were found in a similar concentration range in all exposure scenarios, despite large differences in exposure concentrations: In comparison to the HVO_PM+NOx_ exposure, the Diesel_PM_ exposure contained a 90-fold higher BTEX concentration (710 µg/m^3^ and 7.9 µg/m^3^, respectively).

Only 4-MHA was increased after exposure (t = 3 h) to Diesel_PM_ exhaust compared to before exposure (t = 0 h). Most other biomarkers decreased after 3 h of exposure (Figure 2A−D and Table S3). This effect was observed independently from the exposure scenario (FA, Salt_PM_, HVO_NOx_, HVO_PM+NOx_, or Diesel_PM_), indicating that participants were exposed to higher concentrations of VOCs from other sources before entering the exposure chamber.

The urinary levels for 3-HPMA, BMA, and PMA (Figure 2 and Table S3) were in the same range as those found in the general population [26], and probably not affected by any of the exposure scenarios (with the exception of 4-MHA). Similar urinary concentrations were reported by other researchers:

3-HMPA was measured in concentrations (median levels) of 759 ng/mL in gasoline station workers and 670 ng/mL in the control group [27]; in average concentrations of 1546 ± 1643 ng/mL (smokers) and 406 ± 487 ng/mL (non-smokers) [28]; and in concentrations (median values) of 1686 ng/mL in women from Cape Town [29]. Acrolein is formed by combustion of petroleum fuels and biodiesel, but it is also present in cigarette smoke and cooked food [26,30]. Acrolein can additionally be generated endogenously during lipid peroxidation and inflammation [30], which makes it difficult to use 3-HPMA as specific exposure biomarker.

BMA has been reported in average concentrations of 16 ± 29 ng/mL in smokers and 15 ± 32 ng/mL in non-smokers [28]; in concentrations (median values) of 15 ng/mL in women in Cape Town [29]; and in average concentrations of 8.7 ng/mL in low exposed jet-fuel workers [31]. PMA was measured in average concentrations of 0.9 ± 2.1 ng/mL (smokers) and 0.6 ± 0.4 ng/mL (non-smokers) [28]; in concentrations (median values) of 0.05 ng/mL in women in Cape Town [29]; and in average concentrations of 0.5 ng/mL in low exposed jet-fuel workers [31]. 4-MHA concentrations were slightly lower in our exposure studies compared to other studies (Figure 2 and Table S3): 3- and 4-MHA were reported in average concentrations of 1020 ± 1379 ng/mL in smokers and 579 ± 3692 ng/mL in non-smokers [28]; and in concentrations (median values) of 1061 ng/mL in women from Cape Town [29]. It is possible that we underestimated xylene exposure in our study, as we analyzed 4-MHA as specific marker for *p*-xylene, while others reported the sum of 3- and 4-MHA, which results from the total exposure of *m*- and *p*-xylene.

BTEX are formed from combustion processes and smoking, but they are also omnipresent contaminants of indoor air [17,32,33]. Air concentrations of BTEX in our exposures were low, with maximal concentrations of 8 µg/m^3^ BTEX during the HVO_PM+NOx_ exposure (Table S1), and thus similar to air concentrations measured in North American (0.27−4.9 µg/m^3^) and European countries (0.02−26 µg/m^3^) [17]. These values are well below the limit values of 100 mg/m^3^, as reported by the Swedish Work Environment Authority [34], and below the concentrations of several mg/m^3^ where toxic effects of most VOCs occur [17].

### 3.3. Effect on Oxidative Stress

Urine samples were analyzed for 8-oxodG, a biomarker of DNA oxidative damage [35], and 4-HNE-MA, an urinary metabolite product of lipid peroxidation [36].

Urinary 8-oxodG was detected in average concentrations ranging from 6.7 to 8.9 ng/mL urine across all samples. No difference was observed between different time points (0 h, 3 h, 24 h), or different exposure scenarios (FA, Salt_PM_, HVO_NOx_, HVO_NOx+PM_, Diesel_PM_) (Figure 2E and Table S3). The fact that no difference in urinary 8-oxodG was observed between 10-year-old samples from the petroleum diesel study and urine samples from the recently performed HVO exposure study suggests that urinary 8-oxodG is a stable biomarker, as reported earlier [37]. Similar concentrations have been measured with LC-MS in other studies, [38,39,40], including an inter-laboratory comparison study [41].

Interaction of ROS with DNA can lead to the production of 8-oxodG, which is considered the major oxidation product in nuclear DNA [35]. 8-oxodG in urine mainly originates from oxidation of deoxyguanosine triphosphate in the nucleotide pool [35]. However, 8-oxodG concentrations are correlated with urinary excretion of 8-hydroxyguanine that is considered to originate from DNA repair processes. As such, urinary excretion of 8-oxodG is considered to be a proxy measure of oxidative damage to DNA [42].

In these short-term exposure studies on HVO and petroleum diesel, no alterations of urinary 8-oxodG were observed. This is in line with previous results that reported no signs of oxidative stress in peripheral blood mononuclear cells (PBMCs) after the same short-term (3 h) exposure study with Diesel_PM_ [43]. However, longer exposure times or higher exposure concentrations could cause an increase in urinary 8-oxodG. A repeated exposure study (3 days) to diesel exhaust inside diesel-powered trains caused DNA strand breaks in PBMCs [44]. A review by Moller et al. compiled several studies that showed associations of air pollution with increased urinary excretion of 8-oxodG [45].

### 3.4. Effect on Lipid Peroxidation and Inflammation

In all exposure scenarios of the HVO exposure study, urinary 4-HNE-MA was found in high concentrations throughout all samples, ranging from 56 to 141 ng/mL urine (Figure 2F and Table S3). After the exposure to Diesel_PM_ exhaust (study performed in 2009–2010), urinary 4-HNE-MA concentrations were 10 times lower (7.7−10 ng/mL), suggesting instability issues with this biomarker. Indeed, instability of 4-HNA-MA in human urine due to spontaneous conversion to 4-hydroxy-2-nonenoic acid lactone has been reported before [46]. Kuiper et al. reported slightly lower values compared to our HVO exposure study, ranging from 7.4 to 225 nM (2.4–72 ng/mL) of 4-HNE-MA in smokers upon the cessation of smoking [46].

In the short-term HVO exposure study, we did not detect any differences in 4-HNE-MA between FA, Salt_PM_, and HVO_NOx_ exhaust at different time points (0 h, 3 h, 24 h), but HVO_PM+NOx_ exposure increased 4-HNE-MA significantly from 64 ng/mL urine (before exposure) to 141 ng/mL (24 h after exposure, *p* < 0.001) (Figure 2F and Table S3). NOx exposure only (HVO_NOx_) did not alter 4-HNE-MA concentrations; therefore, the increase in 4-HNE-MA after HVO_PM+NOx_ was likely to originate from the HVO particle fraction. This is in line with several studies reporting the particle-induced formation of ROS and inflammatory processes [45,47]. Lipid oxidation products, such as 4-HNE, can be formed during those processes of oxidative stress and inflammation [36]. Elevated levels of 4-HNE have been found in the urine of smokers [46], as well as in lung tissue and in breath condensates of patients with chronic obstructive pulmonary disease (COPD) [48].

In addition to the urinary measurements of 4-HNE-MA, the expression of inflammatory-related proteins (N = 92) in plasma samples from 18 participants exposed to FA and HVO_PM+NOx_ exhaust were analyzed using proximity extension assay. No plasma samples were available from the petroleum diesel study. No significant differential expression of any inflammatory proteins was found between the HVO_PM+NOx_ exhaust and FA exposure. Our data suggest that exposure to HVO exhaust was too low to induce effects in inflammatory proteins in plasma from the exposed participants. In future studies, exhaled breaths or nasal lavages of the participants could be analyzed as a more sensitive matrix for inflammatory markers [49].

### 3.5. Total Excretion of Biomarkers

**Figure 3:**
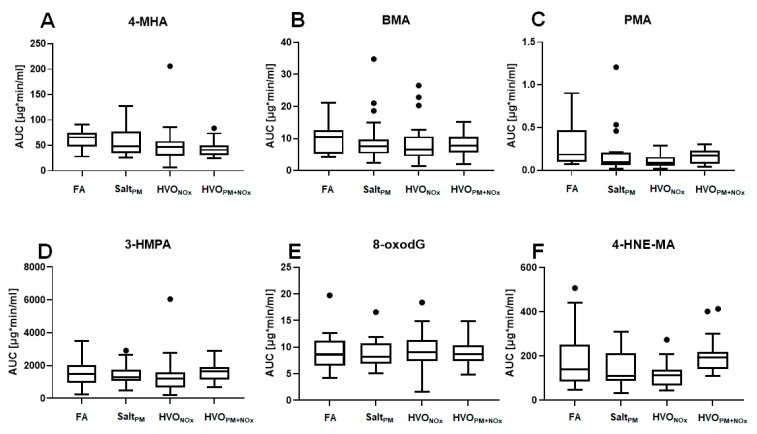
Total excretion of biomarkers, calculated as the area under the excretion curves (AUCs). Box-and-whisker plots display the medians and upper and lower quartiles. Outliers are plotted as individual points. AUCs were calculated for 4-MHA (**A**), BMA (**B**), PMA (**C**), 3-HPMA (**D**), 8-oxodG (**E**), and 4-HNE-MA (**F**), after the following exposure scenarios: filtered air (FA), particle control (Salt_PM_), HVO exhaust from a modern non-road vehicle with aftertreatment system (HVO_NOx_) and without exhaust aftertreatment (HVO_PM+NOx_). Statistical analyses of spot urine samples were performed using Mann–Whitney U test (no significant difference from FA was observed, *p* < 0.05). All data are presented in Table S5.

In the HVO exposure study, we collected all urine from the participants up to 24 h after exposure. The total excreted amount of urinary metabolites in these urine samples was calculated and expressed as the area under the excretion curve (AUC). The AUCs for 4-MHA, BMA, PMA, 3-HPMA, 8-oxodG, and 4-HNE-MA after all exposure scenarios of the HVO exposure study are presented in Figure 3 and Table S4. AUCs for PAH metabolites were not calculated, due to their low urinary concentrations. For the exposure with petroleum diesel, no such calculations were possible, because only spot urine samples were available, whereas in the HVO exposure study, the total excreted volume of urine was collected up to 24 h after exposure.

The AUC of 3-HPMA ranged from 1189 to 1657 µg × min/mL urine (median values), followed by 4-MHA (42–66 µg × min/mL), BMA (6.5–11 µg × min/mL urine) and PMA (0.1–0.2 µg × min/mL urine). The AUC of 8-oxodG ranged from 8.2 to 9.1 µg × min/mL urine. For none of these biomarkers was a difference in AUC observed between different exposure scenarios (FA, Salt_PM_, HVO_NOx_, HVO_PM+NOx_).

The AUC of 4-HNE-MA ranged from 110 to 192 µg × min/mL urine throughout all samples. We observed a non-significant trend of increased AUC after exposure to HVO_PM+NOx_ compared to FA: 192 min × µg/mL (median value) compared to 141 min × µg/mL (median value, *p* > 0.05), respectively (Figure 3 and Table S4). This trend confirmed our observation of increased 4-HNE-MA after 24 h exposure to HVO_PM+NOx_ in spot urine samples (see above).

For all exposure scenarios of the HVO exposure study (including FA and Salt_PM_), the total inhaled amount of measured PAHs and VOCs was compared to the total excreted dose in urine (in nmol) (Table S5). PMA and 4-MU were both found in very low urinary concentrations; therefore, calculations of the total excreted dose are difficult to interpret. For all other measured compounds, the excreted amounts of urinary metabolites were higher than the calculated available dose in the lungs after the short-term (3 h) exposure, independently of the exposure scenario (FA, Salt_PM_, HVO_NOx_, HVO_NOx+PM_). Our data confirmed our earlier observations (above) that PAH and VOC exposure resulted from sources other than the short-term exposure study.

## 4. Conclusions

In this study, we explored biomarkers and putative toxic endpoints for short-term exposures to HVO and petroleum diesel exhaust. To the best of our knowledge, this is the first controlled human exposure study investigating the inhalation of HVO exhaust fumes in humans.

In general, our data showed that exposure biomarkers were in the range found in the general population, suggesting that short-term exposures to HVO exhaust and petroleum diesel exhaust were not sufficient for the detection of specific biomarkers in concentrations above common background levels. After 3 h of exposure, most exposure biomarkers were even slightly decreased. Only 4-MHA, the biomarker for *p*-xylene, increased after 3 h exposure to petroleum diesel. Urinary PAH metabolites were present at very low levels, independently of exposure scenarios and time points (0 h, 3 h, 24 h after exposure).

No alterations in oxidative damage, measured as urinary 8-oxodG, were observed. The biomarker for lipid peroxidation, 4-HNE-MA, was significantly increased 24 h after exposure to HVO_PM+NOx_ compared to the control exposure (FA). In addition, no such increase was observed for HVO_NOx_, suggesting that the particulate fraction or organic compounds of the HVO exhaust were associated with the increase. No significant difference in the expression of inflammatory proteins was found in plasma samples from participants exposed to HVO_PM+NOx_ exhaust and FA.

Our results are in line with Gren et al., who demonstrated that this short-term (3 h) HVO exposure did not impact the participants’ health, reporting only mild self-rated symptoms from the eyes and throat compared to FA exposure [11]. Symptoms were increased after exposure to HVO_PM+NOx_ exhaust from the vehicle without an aftertreatment system, but not after HVO_NOx_ exhaust from the vehicle with an aftertreatment system that efficiently removed volatile organic compounds and particles by number and mass [11]. Additionally, the indication that lipid peroxidation was lower for the exposure to HVO_NOx_ compared to HVO_PM+NOx_ suggests the benefits of implementing such aftertreatment systems.

However, this exposure study only investigated short-term exposure with low exhaust concentrations in healthy people. It remains unknown what effects could be caused by long-term exposure to HVO exhaust, especially in sensitive population groups such as children, older people, or people with underlying health conditions.

## Data Availability

All data is presented in this manuscript and its Supplementary Tables.

## Supplementary Materials

The following are available online at https://www.mdpi.com/article/10.3390/ijerph18126492/s1, Table S1: Summary of the average exposure concentrations and particle characteristics. Full details of exposures and characterization have been described previously for HVO exhaust [11] and petroleum diesel exhaust [12]; Table S2: Analytical details for the quantification of urinary biomarkers. The table includes retention time (RT), quantitative ion transition, collision energy (CE) in electron volt (eV), and limit of detection (LOD). Method details are described in Material and Methods; Table S3: Concentrations of urinary biomarkers after exposure to HVO and petroleum diesel exhaust. Averages and standard deviations of urinary metabolites (density-adjusted) from 18 volunteers after exposure to: filtered air (FA), aerosolized dry NaCl (Salt_PM_), HVO exhaust from a modern non-road vehicle with exhaust aftertreatment (HVO_NOx_) and without exhaust aftertreatment (HVO_PM+NOx_), as well as petroleum diesel exhaust (Diesel_PM_). Statistical analyses were performed using the Wilcoxon signed rank test (* *p* < 0.05, ** *p* < 0.01, *** *p* < 0.001, different from time point 0 h (before exposure)); Table S4: Total excretion of biomarkers after different exposure scenarios. Total excretion of urinary biomarkers, as calculated as area under the excretion curve (AUC), after exposure to filtered air (FA), aerosolized dry NaCl (Salt_PM_), HVO exhaust from a modern non-road vehicle with aftertreatment system (HVO_NOx_) and without exhaust aftertreatment (HVO_PM+NOx_). Average and median values are shown, as well as the 25th to 75th percentile, standard deviation (SD) and coefficient of variation (CV). Statistical analyses were performed using Mann–Whitney U tests (no significant differences from FA were observed, *p* < 0.05); Table S5: Total exposure and total excretion of VOCs. Compared were air concentrations of VOCs (nmol/m^3^), total inhaled amounts of VOCs in the lung (nmol) during 3 h of exposure, and the total excreted urinary amount of VOC metabolites (nmol) up to 24 h after exposure. The sum of 2,3-hydroxyfluorenes is expressed as ∑ OH-Flu, and the sum of 1,2,3,4-hydroxyphenanthrenes is expressed as ∑ OH-Phe.

## Abbreviations

Hydrogenated vegetable oil (HVO); occupational exposure limit (OEL); elemental carbon (EC); particulate matter (PM); nitrogen oxides (NOx); volatile organic compounds (VOCs); polycyclic aromatic hydrocarbons (PAHs); benzene, toluene, ethyl benzene, *m*+*p* xylene and *o*-xylene (BTEX); diesel oxidation catalyst (DOC); diesel particulate filter (DPF); relative humidity (RH); coefficient of variation (CV); retention time (RT); collision energies (CEs); limit of detection (LOD); internal standard (IS); area under the excretion curve (AUC); peripheral blood mononuclear cells (PBMCs); Chronic Obstructive Pulmonary Disease (COPD); 2-naphtol (2-Nap); 2- and 3-hydroxyfluorene (2,3-OH-Flu); 2- and 3-hydroxyphenanthrene (2,3-OH Phe); 1-hydroxyphenanthrene (1-OH-Phe); 4-hydroxy-phenanthrene (4-OH-Phe); 1-hydroxypyrene (1-OH-Pyr), 4-methylhippuric acid (4-MHA); *N*-acetyl-*S*-(benzyl)-L-cysteine (BMA); *N*-acetyl-*S*-(phenyl)-L-cysteine (PMA); 4-methylumbilliferone (4-MU); 3-hydroxypropyl mercapturic acid (3-HPMA); 8-oxo-2’-deoxyguanosine (8-oxodG); 4-hydroxynonenal mercapturic acid (4-HNE-MA)

## References

[1] IARC (2014). Diesel and Gasoline Engine Exhausts and Some Nitroarenes.

[2] European Council (2019). Directive (EU) 2019/130 of the European Parliament and of the Council of 16 January 2019 Amending Directive 2004/37/EC on the Protection of Workers from the Risks Related to Exposure to Carcinogens or Mutagens at Work (Text with EEA Relevance.). Off. J. Eur. Union.

[3] Murtonen T., Aakko-Saksa P., Kuronen M., Mikkonen S., Lehtoranta K. (2010). Emissions with heavy-duty diesel engines and vehicles using FAME, HVO and GTL fuels with and without DOC+ POC aftertreatment. SAE Int. J. Fuels Lubr..

[4] Knothe G. (2010). Biodiesel and renewable diesel: A comparison. Prog. Energy Combust. Sci..

[5] Gren L., Malmborg V.B., Jacobsen N.R., Shukla P.C., Bendtsen K.M., Eriksson A.C., Essig Y.J., Krais A.M., Loeschner K., Shamun S. (2020). Effect of renewable fuels and intake O2 concentration on diesel engine emission characteristics and reactive oxygen species (ROS) formation. Atmosphere.

[6] Gren L., Malmborg V.B., Falk J., Markulad L., Novakovic M., Shamun S., Eriksson A.C., Kristensen T.N., Svenningsson B., Tunér M. (2021). Effects of renewable fuel and exhaust aftertreatment on primary and secondary emissions from a modern heavy-duty diesel engine. J. Aerosol Sci..

[7] Mehus A.A., Reed R.J., Lee V.S., Littau S.R., Hu C., Lutz E.A., Burgess J.L. (2015). Comparison of acute health effects from exposures to diesel and biodiesel fuel emissions. J. Occup. Environ. Med..

[8] Unosson J., Kabele M., Boman C., Nyström R., Sadiktsis I., Westerholm R., Mudway I., Purdie E., Raftis J., Miller M. (2020). Acute cardiovascular effects of controlled exposure to dilute petrodiesel and biodiesel exhaust in healthy volunteers: A crosscover study. Part. Fibre Toxicol..

[9] Moller P., Scholten R.H., Roursgaard M., Krais A.M. (2020). Inflammation, oxidative stress and genotoxicity responses to biodiesel emissions in cultured mammalian cells and animals. Crit. Rev. Toxicol..

[10] Bendtsen K.M., Gren L., Malmborg V.B., Shukla P.C., Tuner M., Essig Y.J., Krais A.M., Clausen P.A., Berthing T., Loeschner K. (2020). Particle characterization and toxicity in C57BL/6 mice following instillation of five different diesel exhaust particles designed to differ in physicochemical properties. Part. Fibre Toxicol..

[11] Gren L., Dierschke K., Mattsson F., Assarsson A., Krais A., Kåredal M., Lovén K., Löndahl J., Pagels J., Strandberg B. (2021). Effects of renewable diesel exhaust on lung function and self-rated symptoms for healthy volunteers in a human chamber exposure study. Part. Fibre Toxicol..

[12] Wierzbicka A., Nilsson P.T., Rissler J., Sallsten G., Xu Y., Pagels J.H., Albin M., Österberg K., Strandberg B., Eriksson A. (2014). Detailed diesel exhaust characteristics including particle surface area and lung deposited dose for better understanding of health effects in human chamber exposure studies. Atmos. Environ..

[13] Alhamdow A., Essig Y.J., Krais A.M., Gustavsson P., Tinnerberg H., Lindh C.H., Hagberg J., Graff P., Albin M., Broberg K. (2020). Fluorene exposure among PAH-exposed workers is associated with epigenetic markers related to lung cancer. Occup. Environ. Med..

[14] Yeh K.C., Kwan K.C. (1978). A comparison of numerical integrating algorithms by trapezoidal, Lagrange, and spline approximation. J. Pharmacokinet. Biopharm..

[15] Assarsson E., Lundberg M., Holmquist G., Bjorkesten J., Thorsen S.B., Ekman D., Eriksson A., Rennel Dickens E., Ohlsson S., Edfeldt G. (2014). Homogenous 96-plex PEA immunoassay exhibiting high sensitivity, specificity, and excellent scalability. PLoS ONE.

[16] Lu S.S., Sobus J.R., Sallsten G., Albin M., Pleil J.D., Gudmundsson A., Madden M.C., Strandberg B., Wierzbicka A., Rappaport S.M. (2014). Are urinary PAHs biomarkers of controlled exposure to diesel exhaust?. Biomarkers.

[17] Li Z., Romanoff L., Bartell S., Pittman E.N., Trinidad D.A., McClean M., Webster T.F., Sjodin A. (2012). Excretion profiles and half-lives of ten urinary polycyclic aromatic hydrocarbon metabolites after dietary exposure. Chem. Res. Toxicol..

[18] Alhamdow A., Zettergren A., Kull I., Hallberg J., Andersson N., Ekstrom S., Berglund M., Wheelock C.E., Essig Y.J., Krais A.M. (2021). Low-level exposure to polycyclic aromatic hydrocarbons is associated with reduced lung function among Swedish young adults. Environ. Res..

[19] Grainger J., Huang W., Patterson D.G., Turner W.E., Pirkle J., Caudill S.P., Wang R.Y., Needham L.L., Sampson E.J. (2006). Reference range levels of polycyclic aromatic hydrocarbons in the US population by measurement of urinary monohydroxy metabolites. Environ. Res..

[20] Vardoulakis S., Giagloglou E., Steinle S., Davis A., Sleeuwenhoek A., Galea K.S., Dixon K., Crawford J.O. (2020). Indoor exposure to selected air pollutants in the home environment: A systematic review. Int. J. Environ. Res. Public Health.

[21] Lim H., Sadiktsis I., de Oliveira Galvao M.F., Westerholm R., Dreij K. (2021). Polycyclic aromatic compounds in particulate matter and indoor dust at preschools in Stockholm, Sweden: Occurrence, sources and genotoxic potential in vitro. Sci. Total Environ..

[22] Masala S., Lim H., Bergvall C., Johansson C., Westerholm R. (2016). Determination of semi-volatile and particle-associated polycyclic aromatic hydrocarbons in Stockholm air with emphasis on the highly carcinogenic dibenzopyrene isomers. Atmos. Environ..

[23] U.S. Environmental Protection Agency (2020). Characterizing Community Exposure to Atmospheric Polycyclic Aromatic Hydrocarbons (PAHs) in The Memphis Tri-State Area: Memphis PAHs Study Final Report.

[24] WHO Regional Office for Europe (2000). Air Quality Guidelines for Europe.

[25] ATSDR (2005). Toxicological Profile for Naphthalene, 1-Methylnaphthalene and 2-Methylnapthalene.

[26] Li A.J., Pal V.K., Kannan K. (2021). A review of environmental occurrence, toxicity, biotransformation and biomonitoring of volatile organic compounds. Environ. Chem. Ecotoxicol..

[27] Frigerio G., Mercadante R., Polledri E., Missineo P., Campo L., Fustinoni S. (2019). An LC-MS/MS method to profile urinary mercapturic acids, metabolites of electrophilic intermediates of occupational and environmental toxicants. J. Chromatogr. B Anal. Technol. Biomed. Life Sci..

[28] Alwis K.U., Blount B.C., Britt A.S., Patel D., Ashley D.L. (2012). Simultaneous analysis of 28 urinary VOC metabolites using ultra high performance liquid chromatography coupled with electrospray ionization tandem mass spectrometry (UPLC-ESI/MSMS). Anal. Chim. Acta.

[29] Everson F., De Boever P., Nawrot T.S., Goswami N., Mthethwa M., Webster I., Martens D.S., Mashele N., Charania S., Kamau F. (2019). Personal NO_2_ and volatile organic compounds exposure levels are associated with markers of cardiovascular risk in women in the Cape Town region of South Africa. Int. J. Environ. Res. Public Health.

[30] Stevens J.F., Maier C.S. (2008). Acrolein: Sources, metabolism, and biomolecular interactions relevant to human health and disease. Mol. Nutr. Food Res..

[31] B’Hymer C., Krieg E., Cheever K.L., Toennis C.A., Clark J.C., Kesner J.S., Gibson R., Butler M.A. (2012). Evaluation and comparison of urinary metabolic biomarkers of exposure for the jet fuel JP-8. J. Toxicol. Environ. Health A.

[32] Bolden A.L., Kwiatkowski C.F., Colborn T. (2015). New Look at BTEX: Are Ambient Levels a Problem?. Environ. Sci. Technol..

[33] Soleimani E. (2020). Benzene, toluene, ethylbenzene, and xylene: Current analytical techniques and approaches for biological monitoring. Rev. Anal. Chem..

[34] AFS (2018). Konsekvensbeskrivning till Föreskrifterna om Hygieniska Gränsvärden.

[35] Moller P., Loft S. (2010). Oxidative damage to DNA and lipids as biomarkers of exposure to air pollution. Environ. Health Perspect..

[36] Dalleau S., Baradat M., Gueraud F., Huc L. (2013). Cell death and diseases related to oxidative stress: 4-hydroxynonenal (HNE) in the balance. Cell Death Differ..

[37] Cooke M.S., Olinski R., Loft S., European Standards Committee on Urinary (DNA) Lesion Analysis (2008). Measurement and meaning of oxidatively modified DNA lesions in urine. Cancer Epidemiol. Biomark. Prev..

[38] Lam P.M., Mistry V., Marczylo T.H., Konje J.C., Evans M.D., Cooke M.S. (2012). Rapid measurement of 8-oxo-7,8-dihydro-2′-deoxyguanosine in human biological matrices using ultra-high-performance liquid chromatography-tandem mass spectrometry. Free Radic. Biol. Med..

[39] Andreoli R., Mutti A., Goldoni M., Manini P., Apostoli P., De Palma G. (2011). Reference ranges of urinary biomarkers of oxidized guanine in (2′-deoxy)ribonucleotides and nucleic acids. Free Radic. Biol. Med..

[40] Lee K.F., Chung W.Y., Benzie I.F. (2010). Urine 8-oxo-7,8-dihydro-2′-deoxyguanosine (8-oxodG), a specific marker of oxidative stress, using direct, isocratic LC-MS/MS: Method evaluation and application in study of biological variation in healthy adults. Clin. Chim. Acta.

[41] Barregard L., Moller P., Henriksen T., Mistry V., Koppen G., Rossner P., Sram R.J., Weimann A., Poulsen H.E., Nataf R. (2013). Human and methodological sources of variability in the measurement of urinary 8-oxo-7,8-dihydro-2′-deoxyguanosine. Antioxid. Redox Signal..

[42] Loft S., Danielsen P., Lohr M., Jantzen K., Hemmingsen J.G., Roursgaard M., Karotki D.G., Moller P. (2012). Urinary excretion of 8-oxo-7,8-dihydroguanine as biomarker of oxidative damage to DNA. Arch. Biochem. Biophys..

[43] Hemmingsen J.G., Moller P., Jantzen K., Jonsson B.A., Albin M., Wierzbicka A., Gudmundsson A., Loft S., Rissler J. (2015). Controlled exposure to diesel exhaust and traffic noise: Effects on oxidative stress and activation in mononuclear blood cells. Mutat. Res..

[44] Andersen M.H.G., Frederiksen M., Saber A.T., Wils R.S., Fonseca A.S., Koponen I.K., Johannesson S., Roursgaard M., Loft S., Moller P. (2019). Health effects of exposure to diesel exhaust in diesel-powered trains. Part. Fibre Toxicol..

[45] Moller P., Danielsen P.H., Karottki D.G., Jantzen K., Roursgaard M., Klingberg H., Jensen D.M., Christophersen D.V., Hemmingsen J.G., Cao Y. (2014). Oxidative stress and inflammation generated DNA damage by exposure to air pollution particles. Mutat. Res. Rev. Mutat. Res..

[46] Kuiper H.C., Langsdorf B.L., Miranda C.L., Joss J., Jubert C., Mata J.E., Stevens J.F. (2010). Quantitation of mercapturic acid conjugates of 4-hydroxy-2-nonenal and 4-oxo-2-nonenal metabolites in a smoking cessation study. Free Radic. Biol. Med..

[47] Lodovici M., Bigagli E. (2011). Oxidative stress and air pollution exposure. J. Toxicol..

[48] Rahman I., van Schadewijk A.A., Crowther A.J., Hiemstra P.S., Stolk J., MacNee W., De Boer W.I. (2002). 4-Hydroxy-2-nonenal, a specific lipid peroxidation product, is elevated in lungs of patients with chronic obstructive pulmonary disease. Am. J. Respir. Crit. Care Med..

[49] Grob N.M., Aytekin M., Dweik R.A. (2008). Biomarkers in exhaled breath condensate: A review of collection, processing and analysis. J. Breath Res..

