# Supplementary material for: Biomarkers after Controlled Inhalation Exposure to Exhaust from Hydrogenated Vegetable Oil (HVO)
Source: Int J Environ Res Public Health. 2021 Jun 16;18(12):6492. doi: 10.3390/ijerph18126492 (PMC8296316; doi:10.3390/ijerph18126492)
Supplement: Supplementary file 1 [file ijerph-18-06492-s001.zip › ijerph-1232560-supplementary.pdf]

Table S1: Summary of the average exposure concentrations and particle characteristics. Full details of exposures and characterization have been described earlier for HVO exhaust [11] and petroleum diesel exhaust [12].

	HVO exposure study [11]				Diesel exposure study [12]	
	FA	$Salt_{PM}$	HVO_{NOx}	HVO_{PM+NOx}	FA	$Diesel_{PM}$
PM_1 (µg/m^3)	<1[a*]	85 ± 13[a]	<1[a*]	93 ± 13[a]	2 ± 2[b]	276 ± 27[b]
Elemental carbon (EC, µg/m^3)	<1	<1	<1	54 ± 6	<1	215 ± 37
NO (ppm)	<0.001	<0.001	2.0 ± 0.1	3.4 ± 0.1	<0.003	9.8 ± 1.0
NO_2 (ppm)	<0.001	<0.001	0.70 ± 0.04	0.57 ± 0.04	<0.002	1.3 ± 0.4
Particle phase PAHs (ng/m^3)	<LOD	<LOD	0.3 ± 0.7	43 ± 3	<LOD	60 ± 12
Gas phase PAHs (ng/m^3)	116 ± 29	96 ± 47	97 ± 11	850 ± 48	<LOD	$7.5 \times 10^3 \pm 0.2 \times 10^3$
Sum BTEX[c] (µg/m^3)	1.3 ± 0.1	1.3 ± 0.1	1.7 ± 0.3	7.9 ± 2.5	<LOD	710 ± 14

[a]Gravimetric mass analysis; [a*]large uncertainties at low mass concentrations. The mass concentrations were in the range of the blank filters (-1 ± 3 µg).
[b]Mass of PM_1 based on TEOM measurements.
[c]Benzene, toluene, ethyl benzene, *m+p* xylene, and *o*-xylene were included in the analysis.

Table S2: Analytical details for quantification of urinary biomarkers. The table includes retention time (RT), quantitative ion transition, collision energies (CE) in electron volt (eV), and limit of detection (LOD). Method details are described in Material and Methods.

Group	Biomarker	RT	Transition	CE (eV)	Internal Standard	RT	Internal Standard	CE (eV)	LOD
PAHs	2-Nap	6.84	143 → 115	-34	D_7-2-Nap	6.79	150 → 122	-34	0.04
	2,3-OH-Flu	7.52	181 → 180	-30	D_8-2,3-OH-Flu	7.48	190 → 188	-30	0.07
	2,3-OH-Phe	7.77	193 → 165	-41	D_9-2,3-OH-Phe	7.68	202 → 174	-41	0.07
	1-OH-Phe	7.89	193 → 165	-41	D_9-1-OH-Phe	7.85	202 → 174	-41	0.07
	4-OH-Phe	8.01	193 → 165	-41	D_9-4-OH-Phe	7.95	202 → 174	-41	0.07
	1-OH-Pyr	8.29	217 → 189	-50	D_9-1-OH-Pyr	8.24	226 → 198	-50	0.07
other VOCs	4-MHA	2.86	192 → 91	-17	D_7-4-MHA	2.84	199 → 98	-17	1.52
	BMA	3.72	252→ 123	-20	D_4-BMA	3.73	257→ 128	-20	0.03
	PMA	3.65	238 → 109	-22	D_5-PMA	3.66	243 → 114	-22	0.02
	4-MU	3.83	175 → 147	-30	$^{13}C_4$-4-MU	3.82	179 → 150	-30	0.07
	3-HPMA	2.41	220 → 91	-17	D_6-3-HPMA	2.40	226 → 97	-17	0.08
ox stress Inflamm.	4-HNE-MA	2.96	318 → 171	-28					
	8-oxodG	7.52	284 → 168	17	$^{15}N_5$-8-oxodG	7.48	289 → 173	17	0.12

Table S3: Concentrations of urinary biomarkers after exposure to HVO and petroleum diesel exhaust. Average and standard deviation of urinary metabolites (density adjusted) from 18 volunteers after exposure to: filtered air (FA), aerosolized dry NaCl (Salt$_{PM}$), HVO exhaust from a modern non-road vehicle with exhaust aftertreatment (HVO$_{NOx}$) and without exhaust aftertreatment (HVO$_{PM+NOx}$), as well as petroleum diesel exhaust (Diesel$_{PM}$). Statistical analysis was performed using Wilcoxon signed rank test (*P < 0.05, **P < 0.01, ***P < 0.001, different from time point 0 h (before exposure)).

Group	Biomarker	Time point	FA	Salt $_{PM}$	HVO $_{Nox}$	HVO $_{PM+NOx}$	Diesel $_{PM}$
			average ± stdv				
PAHs	2-Nap	0 h	3.5 ± 3.2	3.6 ± 3.1	2.7 ± 2.5	2.8 ± 2.6	3.4 ± 3.9
		3 h	2.3 ± 2.2	2.1 ± 1.6	1.6 ± 1.0	1.8 ± 1.5	2.7 ± 3.4
		24 h	2.8 ± 2.6	2.8 ± 1.7	2.8 ± 2.2	3.9 ± 7.1	3.1 ± 2.9
	2,3-OH-Flu	0 h	0.2 ± 0.2	0.2 ± 0.4	0.1 ± 0.1	0.2 ± 0.1	0.2 ± 0.3
		3 h	0.2 ± 0.1	0.1 ± 0.1	0.1 ± 0.1	0.1 ± 0.1	0.2 ± 0.1
		24 h	0.2 ± 0.1	0.1 ± 0.1	0.2 ± 0.1	0.1 ± 0.1	0.2 ± 0.1
	2,3-OH-Phe	0 h	0.2 ± 0.2	0.2 ± 0.2	0.1 ± 0.1	0.2 ± 0.2	0.2 ± 0.2
		3 h	0.2 ± 0.1	0.1 ± 0.1	0.1 ± 0.1	0.1 ± 0.1	0.1 ± 0.1
		24 h	0.2 ± 0.2	0.2 ± 0.1	0.2 ± 0.2	0.1 ± 0.1	0.2 ± 0.2
	1-OH-Phe	0 h	0.3 ± 0.2	0.4 ± 0.5	0.2 ± 0.2	0.2 ± 0.3	0.3 ± 0.2
		3 h	0.2 ± 0.1	0.3 ± 0.4	0.2 ± 0.2	0.2 ± 0.2	0.3 ± 0.2
		24 h	0.3 ± 0.3	0.4 ± 0.5	0.3 ± 0.3	0.2 ± 0.1	0.3 ± 0.2
	4-OH-Phe	0 h	0.0 ± 0.0	0.0 ± 0.1	0.0 ± 0.1	0.0 ± 0.0	0.0 ± 0.0
		3 h	0.0 ± 0.0	0.0 ± 0.0	0.0 ± 0.0	0.0 ± 0.0	0.0 ± 0.0
		24 h	0.0 ± 0.0	0.0 ± 0.1	0.0 ± 0.0	0.0 ± 0.0	0.0 ± 0.0
	1-OH-Pyr	0 h	0.1 ± 0.0	0.1 ± 0.1	0.1 ± 0.0	0.1 ± 0.0	0.1 ± 0.1
		3 h	0.1 ± 0.0	0.0 ± 0.0	0.0 ± 0.0	0.0 ± 0.0	0.1 ± 0.0
		24 h	0.1 ± 0.0	0.1 ± 0.0	0.1 ± 0.1	0.1 ± 0.0	0.1 ± 0.1
other VOCs	4-MHA	0 h	59 ± 28	78 ± 177	44 ± 24	31 ± 11	79 ± 55
		3 h	34 ± 13***	57 ± 75	27 ± 14***	23 ± 8.0*	118 ± 64***
		24 h	51 ± 21	63 ± 113	56 ± 64	36 ± 30	77 ± 74
	BMA	0 h	8.8 ± 5.8	11 ± 16	8.9 ± 6.5	8.1 ± 6.4	10 ± 6.9
		3 h	7.2 ± 6.0***	6.1 ± 4.2**	5.3 ± 4.4***	5.2 ± 2.9**	9.5 ± 9.3
		24 h	9.5 ± 5.7	9.6 ± 7.8	9.7 ± 7.3	7.1 ± 3.5	12 ± 7.6
	PMA	0 h	0.2 ± 0.2	0.1 ± 0.1	0.1 ± 0.1	0.1 ± 0.0	0.1 ± 0.0
		3 h	0.1 ± 0.1*	0.2 ± 0.3	0.0 ± 0.1***	0.0 ± 0.0	0.0 ± 0.0***
		24 h	0.2 ± 0.2	0.2 ± 0.2	0.1 ± 0.1	0.1 ± 0.1*	0.1 ± 0.0
	4-MU	0 h	0.4 ± 0.5	1.0 ± 1.4	0.5 ± 0.8	0.9 ± 1.6	0.7 ± 0.9
		3 h	0.3 ± 0.3	1.0 ± 1.1	0.3 ± 0.3	0.6 ± 1.0	0.4 ± 0.4
		24 h	0.6 ± 1.0	1.6 ± 1.8	0.5 ± 0.5	0.7 ± 1.0	0.5 ± 0.5
	3-HMPA	0 h	1564 ± 1165	1281 ± 1202	1400 ± 957	911 ± 618	958 ± 842
		3 h	832 ± 838**	700 ± 482*	649 ± 612***	650 ± 371*	533 ± 225***
		24 h	1307 ± 969	1385 ± 906	1073 ± 558	1366 ± 567*	958 ± 622
ox. Stress / inflammation	4-HNE-MA	0 h	65 ± 76	68 ± 70	44 ± 36	64 ± 71	9.1 ± 10
		3 h	70 ± 44	96 ± 62	61 ± 35	75 ± 37	7.7 ± 5.3
		24 h	58 ± 44	56 ± 56	57 ± 52	141 ± 103***	10 ± 8.2
	8-oxodG	0 h	8.2 ± 3.8	9.0 ± 3.6	8.9 ± 3.8	8.2 ± 3.6	7.7 ± 2.6
		3 h	7.0 ± 2.6	8.0 ± 3.6	7.6 ± 3.1	7.1 ± 2.8	6.7 ± 2.3
		24 h	8.5 ± 3.2	8.9 ± 3.2	8.7 ± 4.0	7.8 ± 3.0	7.5 ± 2.1

Table S4: Total excretion of biomarkers after different exposure scenarios.
Total excretion of urinary biomarkers, as calculated as area under the excretion curve (AUC), after exposure to filtered air (FA), aerosolized dry NaCl ($Salt_{PM}$), HVO exhaust from a modern non-road vehicle with aftertreatment system (HVO_{NOx}) and without exhaust aftertreatment (HVO_{PM+NOx}). Shown are average and median values, as well as 25th-75th percentile, standard deviation (SD) and coefficient of variation (CV). Statistical analysis was performed using Mann-Whitney U test (no significant difference to FA were observed, $P < 0.05$).

	Exposure	Average [µg*min/mL]	Median [µg*min/mL]	25th percentile [µg*min/mL]	75th percentile [µg*min/mL]	SD [µg*min/mL]	CV [%]
4-HMA	FA	147	66	50	76	351	239
	$Salt_{PM}$	101	51	36	78	188	187
	HVO_{NOx}	56	51	33	59	42	75
	HVO_{PM+NOx}	78	42	31	50	144	184
BMA	FA	10	11	5,4	12	4,9	49
	$Salt_{PM}$	9,8	7,5	5,4	9,6	7,7	78
	HVO_{NOx}	8,8	6,5	4,6	9,4	6,8	77
	HVO_{PM+NOx}	8,0	7,7	6,1	10	3,4	42
PMA	FA	0,3	0,2	0,1	0,4	0,2	81
	$Salt_{PM}$	0,2	0,1	0,1	0,2	0,3	141
	HVO_{NOx}	0,1	0,1	0,1	0,1	0,1	70
	HVO_{PM+NOx}	0,2	0,2	0,1	0,2	0,1	47
4-MU	FA	1,0	0,6	0,4	1,2	1,1	101
	$Salt_{PM}$	1,8	1,0	0,6	2,4	1,9	102
	HVO_{NOx}	0,8	0,9	0,5	1,2	0,5	58
	HVO_{PM+NOx}	1,1	0,5	0,3	1,0	1,4	126
3-HPMA	FA	1612	1497	1008	1942	782	49
	$Salt_{PM}$	1472	1268	1111	1684	648	44
	HVO_{NOx}	1472	1189	748	1584	1263	86
	HVO_{PM+NOx}	1600	1657	1167	1866	564	35
4-HNE-MA	FA	181	141	88	235	130	72
	$Salt_{PM}$	146	110	91	183	87	60
	HVO_{NOx}	113	112	68	133	56	50
	HVO_{PM+NOx}	203	192	145	211	85	42
8-oxodG	FA	9,2	8,6	6,9	11,1	3,5	38
	$Salt_{PM}$	8,9	8,2	6,9	10,4	2,7	30
	HVO_{NOx}	9,4	9,1	7,5	10,8	3,7	39
	HVO_{PM+NOx}	9,0	8,7	7,5	10,0	2,4	27

Table S5: Total exposure and total excretion of VOCs. Compared were air concentrations of VOCs ($nmol/m^3$), total inhaled amount of VOCs in the lung (nmol) during 3 h of exposure, and the total urinary excreted amount of VOC metabolites (nmol) up to 24 h after exposure. The sum of 2,3-hydroxyfluorenes is expressed as ∑ OH-Flu; and the sum of 1,2,3,4-hydroxyphenanthrenes is expressed as ∑ OH-Phe.

Exposure	Total Inhaled Amount (nmol)						
	Nap	Flu	Phe	Pyr	p-xylene	toluene	benzene
FA	1.1	0.2	0.1	0.0	11,97	8.5	3.6
$Salt_{PM}$	0.6	0.2	0.1	0.0	10,19	10.0	5.9
HVO_{NOx}	0.7	0.1	0.1	0.0	9,82	11.6	22.8
HVO_{PM+NOx}	11.0	0.1	0.1	0.0	11,04	37.2	208
**Exposure**	**Total Excreted Amount (nmol)**						
	2-OH Nap	∑ OH-Flu	∑ OH-Phe	1-OH Pyr	4-MHA	BMA	PMA
FA	23.1	1.25	1.85	0.35	783	43.4	1.4
$Salt_{PM}$	21.8	1.07	1.68	0.30	522	37.4	1.2
HVO_{NOx}	19.9	0.84	1.32	0.33	336	32.5	0.4
HVO_{PM+NOx}	21.9	1.04	1.35	0.31	899	35.8	0.7
**Exposure**	**% (Total Excreted Amount/ Total Inhaled Amount)**						
	Nap	Flu	Phe	Pyr	p-xylene	toluene	benzene
FA	2176	740	1650	n.d.	6536	512	38
$Salt_{PM}$	3670	634	1663	n.d.	5124	374	20
HVO_{NOx}	2894	638	1565	n.d.	3426	281	1.9
HVO_{PM+NOx}	199	754	1005	n.d.	8141	96.2	0.3
